# Microstructural meal pattern analysis reveals a paradoxical acute increase in food intake after nicotine despite its long-term anorexigenic effects

**DOI:** 10.1007/s00213-022-06071-2

**Published:** 2022-02-07

**Authors:** Kokila Shankar, Frederic Ambroggi, Olivier George

**Affiliations:** 1 Department of Neuroscience, The Scripps Research Institute, 10550 N. Torrey Pines Road, La Jolla, CA 92037 USA; 2 Department of Psychiatry, School of Medicine, University of California San Diego, La Jolla, CA 92093 USA; 3 Laboratoire de Neurosciences Cognitives, CNRS, Aix-Marseilles Université, 3, place Victor-Hugo, 13331 cedex 3 Marseille, France

**Keywords:** Self-administration, Metabolism, Addiction, Feeding, Food, Drinking

## Abstract

**Rationale:**

Nicotine consumption in both human and animal studies has been strongly associated with changes in feeding-related behaviors and metabolism. The current dogma is that nicotine is an anorexic agent that decreases food intake and increases metabolism, leading to decreased body weight gain. However, there are conflicting reports about the acute effects of nicotine on hunger in humans. No study has reported nicotine-induced decreases in food intake within minutes of consumption, suggesting that our understanding of the pharmacological effects of nicotine on appetite and feeding may be incorrect.

**Objectives:**

The aim of this study was to elucidate effects of acute nicotine intake on feeding and drinking behavior.

**Methods:**

Adult male Wistar rats were trained to intravenously self-administer nicotine. Microstructural and macrostructural behavioral analyses were employed to look at changes in food and water intake at different timescales.

**Results:**

At the macrostructural level (hours to days), nicotine decreased body weight gain, decreased feeding, and was associated with increases in feeding and body weight gain during abstinence. At the microstructural level (seconds to minutes), nicotine increased feeding and drinking behavior during the first 5 min after nicotine self-administration. This effect was also observed in animals that passively received nicotine, but the effect was not observed in animals that self-administered saline or passively received saline.

**Conclusions:**

These results challenge the notion that the initial pharmacological effect of nicotine is anorexigenic and paradoxically suggest that an acute increase in food intake minutes after exposure to nicotine may contribute to the long-term anorexigenic effects of nicotine.

## Introduction

Although cigarette smoking and tobacco use have been primarily reported to occur because of the psychoactive properties of nicotine, robust evidence suggests that tobacco is also used for its effects on body weight. Smokers exhibit lower body weight gain compared with nonsmokers, and smoking cessation leads to a robust weight gain that contributes to relapse (Audrain-McGovern & Benowitz 2011; Filozof et al. 2004; Komiyama et al. 2013).

Chronic exposure to nicotine or tobacco smoke in humans and nonhuman animals reduces body weight and food intake and increases metabolism, contributing to the decrease in body weight (L. L. Bellinger et al. 2010; Grebenstein et al. 2013; Grunberg et al. 1986; Hellerstein et al. 1994; Rupprecht et al. 2018). The dogma for the past 30 years is that these effects are produced by the acute activation of nicotinic receptors in the brain and body (Calarco & Picciotto 2020; Govind et al. 2009, 2012). Preclinical and clinical models have consistently shown that nicotine decreases food intake hours and days after initiating chronic nicotine exposure (Bishop et al. 2002; McNair & Bryson 1983; Mineur et al. 2011; O’Dell et al. 2007; Winders & Grunberg 1990; Zachari et al. 2016); however, these studies did not report the initial (acute) effect of nicotine on feeding. Additionally, no studies have reported nicotine-induced decreases in food intake when given acutely in humans, and one study even reported an increase in caloric intake after acute nicotine exposure in humans (Perkins 1992; Perkins et al. 1991).

Understanding the acute effects of nicotine intake is important because of nicotine’s rapid pharmacokinetic profile. Nicotine reaches the brain in ~ 7 s after a single cigarette puff (Benowitz et al. 2009), and brain nicotinic receptors are saturated after only three puffs (Rose et al. 2010). Furthermore, most nicotinic receptors desensitize in minutes, suggesting that if activation of nicotinic receptors produces the anorectic properties of nicotine, then nicotine self-administration should produce a robust decrease in hunger or caloric intake within seconds to minutes. However, studies to date have not evaluated the effects of nicotine on feeding at the microstructural level (i.e., seconds to minutes after exposure to nicotine).

Microstructural meal pattern analysis is a technique that is commonly used in feeding behavior studies that can be employed to analyze acute behavioral changes (Davis et al. 1999; Grigson et al. 1993). This approach measures behavioral changes within a small timescale, usually seconds to minutes, as a response to a specific singular event. For example, microstructural meal pattern analysis shows that the acute administration of cocaine, a potent anorectic drug, decreases food intake in rats (Cooper & van der Hoek 1993). To date, only one study by Romero et al. has used microstructural analysis to analyze components of feeding behavior in relation to nicotine intake; their study focused on the reinforcing effects on nicotine on food-seeking behavior (Romero et al. 2018). However, there are currently no studies comparing the effects of nicotine on acute vs. long-term feeding behavior.

To address this gap in the literature and identify the acute and long-term effects of nicotine on caloric intake, feeding behavior, and body weight, we performed both microstructural and macrostructural behavioral analyses of rats that intravenously self-administered nicotine. The self-administration paradigm was used as it yields pharmacokinetic distribution of nicotine that is most consistent with human use, and long-access paradigms can mimic more effectively the consistent smoking behaviors seen in humans (Corrigall & Coen 1989; LeSage et al. 2003; Valentine et al. 1997). Macrostructural analyses examined the effects of chronic nicotine intake on feeding, drinking, and body weight over 7 weeks of access to intravenous nicotine self-administration (23 h/day, 4 days per week), while microstructural analyses examined the changes in food and water self-administration in seconds to minutes following each nicotine self-administration event using peristimulus time histograms. Based on the current dogma, we hypothesized that acute and chronic nicotine self-administration decreases caloric intake, feeding, and body weight.

## Methods

### Animals

Adult male Wistar rats (*N* = 17, 8–10 weeks, 250–275 g at the start of the study; Charles River, Hollister, CA, USA) were used for the experiments. The animals were group-housed and maintained on a 12 h/12 h light/dark cycle (lights off at 10:00 AM) with ad libitum access to food (45 mg grain-based tablets, TestDiet, St. Louis, MO, USA) and tap water. Body weights of all animals were recorded daily throughout the study. All of the animal procedures were approved by The Scripps Research Institute Institutional Animal Care and Use Committee and were in accordance with the National Institutes of Health guidelines.

### Drugs

Nicotine hydrogen tartrate salt (nicotine bitartrate, Sigma, Natick, MA, USA) was dissolved in 0.9% saline (pH 7.4) and self-administered via an indwelling intravenous jugular catheter (0.03 mg/kg/100 µL infusion). Nicotine salt is preferred to the free base form for more stable storage and use during the experiments. Doses reported in this study are expressed as the free base concentrations.

### Nicotine self-administration

The apparatus and detailed procedures for both intravenous catheterization and nicotine self-administration have been described in detail previously (George et al. 2007; O’Dell & Koob 2007). Briefly, rats were anesthetized with an isoflurane/oxygen vapor mixture (1–5%). Intravenous catheters connected to an external cannula were implanted into the rats’ jugular veins. All animals were allowed to recover for 5–7 days. Catheters were flushed daily with 0.2 mL of sterile physiological saline containing heparin (30 USP units/mL) and the antibiotic cefazolin. Catheter patency was tested using 0.1 mL of the ultra-short-acting barbiturate Brevital sodium; animals with patent catheters exhibited pronounced loss of muscle tone within seconds of the intravenous injection.

The experiments were conducted at the start of the dark cycle in operant conditioning boxes (MedAssociates, Inc., St. Albans, VT, USA) containing an active lever and cue light (associated with nicotine delivery), an inactive lever associated with no outcomes, a nosepoke associated with food pellet dispensation, a nosepoke associated with water dispensation, and a house light (turning on 12 h into the session, at the start of the light cycle). The rats were first trained to nosepoke for food and water in 23-h sessions before and after recovery from the surgical implantation of jugular catheters but were not given access to the active lever that was associated with nicotine delivery or the inactive lever. Both food and water nosepokes were operated on a FR1 schedule with no timeout period. Each food nosepoke produced one 45 mg standard grain-based chow pellet (TestDiet, St. Louis, MO, USA). Each water nosepoke produced 100 µL of tap water. Following the acquisition of these operant responses, the active and inactive levers were extended, and the rats (*n* = 8) were allowed to self-administer nicotine (0.03 mg/kg/100 µL/1 s, free base, fixed-ratio 1 [FR1], timeout [TO] 20 s) by pressing the active lever. The rats were first given access to nicotine for 1 h per day during the dark cycle (beginning at 10:00 AM) for 1 week and then long access (LgA; 23 h/day) to nicotine for 2 weeks. Following this, animals were placed on a 3-day OFF, 4-day ON intermittent cycle for 6 weeks where animals alternated between 3 sessions in their home cage with no access to nicotine and 4 days of 23-h self-administration sessions for nicotine. Another group of animals (*n* = 9) was also trained to self-administer 0.9% saline solution as a control, following the same paradigm. At all times during these sessions, animals had ad libitum access to food and water through the associated nosepokes. Inactive lever-pressing data was recorded as a measure of general, nonspecific activity within the operant chambers.

All self-administration analyses were conducted during the first 23-h self-administration session following the first 3-day OFF period in the home cage. Passive administration of nicotine or saline was conducted using the MedPC program (MedAssociates, Inc., St. Albans, VT, USA) prior to the start of nicotine self-administration, in which one saline infusion, one nicotine infusion, or three nicotine infusions were administered to the animals in a Latin square design over 6 days. Animals were given access to food and water in the operant chambers but not given access to the active lever.

### Peristimulus time histogram generation

The total food, water, and drug self-administration events and the total active and inactive lever presses for each animal during each day of the experiment were extracted from MedPC and stored as .txt files. A nicotine event comprises 1 active lever press which produces a nicotine infusion (additional lever presses during the 20-s timeout period are not counted as drug events). A food event comprises 1 nosepoke which dispenses one 45 mg food pellet. A water event comprises 1 nosepoke which produces 100 µL of water. The files were imported into Microsoft Excel and then batch imported into Spike2 (Cambridge Electronic Design, Cambridge, United Kingdom).

To generate peristimulus time histograms (PSTH), the normalized probability of intake behavior was plotted against the time surrounding the intake event of interest. First, the total number of events of a single category (drug, food, or water intake) that occurred in 10 s was calculated. This was repeated for each 10-s bin in the 1000-s time window that surrounded a single drug, food, or water self-administration event (intake event of interest). Finally, the number of events in each 10-s bin was divided by the total intake during the 23-h session to identify the probability of behaviors surrounding a specific event and limit the confounding effects of total behavioral output. For example, in comparing food vs. nicotine events, for each nicotine event occurring during the 23-h session, the total number of food events per 10-s bin was measured 1000 s before and after each nicotine event. For each animal, these values were averaged across all events during the session. Finally, those values were then divided by the total number of food events during the session to generate the normalized probability of food events surrounding a nicotine event. The resulting graph generated shows the average normalized probability for all animals during the 23-h self-administration session.

To calculate the duration of feeding and drinking bouts, PSTH were first generated to compare food intake events in a 1000-s window surrounding a single food intake event (food vs. food) and water intake events in a 1000-s window surrounding a single water intake event (water vs. water), respectively. The first derivative (change in *y*-axis over change in *x*-axis of the tangent line to a point on the curve) was calculated for each point on the PSTH curve. On either side of time *t* = 0, the times at which the first derivative equals 0 were identified. The duration of the bout was calculated as the absolute value of the difference between the two times.

### Statistical analysis

All of the data was analyzed using Prism 8 software (GraphPad, San Diego, CA, USA). The peristimulus time histogram data were analyzed between subjects using non-parametric Mann–Whitney (when comparing 2 groups) or Kruskal–Wallis with Dunn’s multiple comparisons (when comparing 3 groups) test as they violated assumptions of normality using the Kolmogorov–Smirnov test. Feeding and drinking bout duration and inactive lever presses were analyzed either by Student’s *t*-test when comparing 2 groups or one-way ANOVA followed by Tukey’s multiple comparison post hoc test when comparing 3 groups. Food intake, water intake, and body weight data were analyzed using two-way ANOVA followed by Sidak (body weight) or Tukey’s (food/water intake) multiple-comparison post hoc test. The data are expressed as mean ± SEM unless otherwise specified.

## Results

### Macrostructural analysis of feeding behavior during nicotine self-administration

We first examined the changes in feeding macrostructure relative to nicotine intake. As this has been studied extensively in the literature, we hypothesized that long-term nicotine intake would reduce body weight gain and food intake. To evaluate the long-term effect of nicotine self-administration on body weight and food and water intake, the rats were given extended access to nicotine self-administration for 6 weeks, during which they received 4 days of 23-h nicotine self-administration (ON) and 3 days without nicotine or saline (OFF) in their home cages with ad lib access to food and water. Two-way ANOVA was used to compare the effects of time and treatment and time × treatment interactions between nicotine self-administration animals and saline animals.

**Fig. 1:**
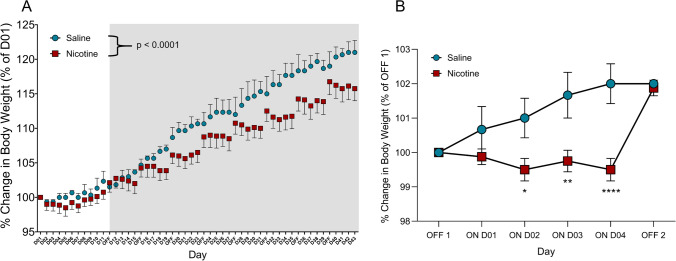
Changes in body weight in nicotine vs. saline rats. **A** Average change in body weight in nicotine (red) and saline (blue) rats over the course of the nicotine self-administration paradigm. Unshaded area represents continuous long access. Gray shaded area represents intermittent long access (4 days ON, 3 days OFF). The data are expressed as a percent change in body weight relative to baseline. Time × treatment interaction, *p* < 0.0001 (two-way repeated-measures ANOVA). **B** Average change in body weight in nicotine (red) and saline (blue) rats within each cycle of the nicotine self-administration paradigm. The data are expressed as a percent change in body weight relative to D01 OFF1. Time × treatment interaction, *p* = 0.0023 (two-way repeated-measures ANOVA). Nicotine (*N* = 8), saline (*N* = 3). *p < 0.05, **p < 0.01, ***p < 0.001, ****p < 0.0001 (Sidak multiple-comparison post hoc test)

A significant increase in body weight was observed throughout the experiment (main effect of time, *F*_50,300_ = 85.73, *p* < 0.0001). Nicotine self-administration decreased body weight gain compared with saline self-administration (time × treatment interaction, *F*_50,300_ = 2.683, *p* < 0.0001; Fig. 1A). The post hoc analysis showed a significant decrease in body weight in nicotine animals compared with saline animals following day 15 of the self-administration paradigm, which is when the animals started the intermittent access phase of the paradigm. This decrease persisted until the end of the experiment.

The effect of nicotine on body weight was further examined after averaging every cycle of the intermittent access paradigm. During the self-administration (ON) phase of the cycle, nicotine animals exhibited a significant decrease in weight gain (time × treatment interaction, *F*_5,30_ = 4.846, *p* = 0.0023; main effect of time, *F*_5,30_ = 7.313, *p* = 0.0001). The post hoc analysis showed that nicotine animals exhibited a decrease in body weight gain on days 3 and 4 compared with saline animals. However, this decrease in body weight was abolished during the abstinence (OFF) phase of the cycle; by the end of the abstinence phase, the relative body weight gain was not significantly different between nicotine and saline animals (Fig. 1B).

**Fig. 2:**
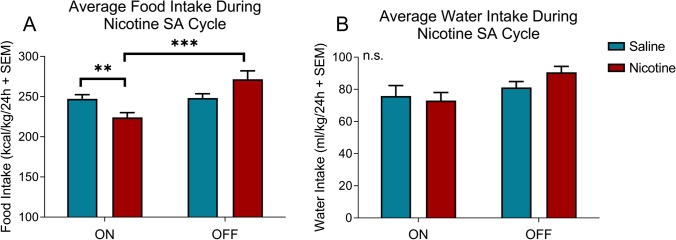
Average food and water intake in nicotine vs. saline rats. **A** Average daily food intake in nicotine (red) vs. saline (blue) rats during the nicotine ON and OFF phases. The data are expressed as calories consumed normalized to body weight in a 24-h period. Time × treatment interaction, *p* = 0.0019 (two-way ANOVA). **B** Average daily water intake in nicotine (red) vs. saline (blue) rats during the nicotine ON and OFF phases. The data are expressed as milliliters of water consumed normalized to body weight in a 24-h period. Time × treatment interaction, *p* = 0.2225 (two-way ANOVA). Nicotine (*N* = 8), saline (*N* = 9). *p < 0.05, **p < 0.01, ***p < 0.001, ****p < 0.0001 (Tukey’s multiple-comparison post hoc test)

The animals were tested on standard chow and tap water to investigate changes in food and water intake across the self-administration paradigm. Two-way ANOVA was used to compare differences in food and water intake between nicotine and saline animals during the self-administration (ON) and abstinence (OFF) phases. Nicotine animals exhibited a significant increase in the total daily chow intake during the abstinence phase compared with saline animals (time × treatment interaction, *F*_1,30_ = 11.53, *p* = 0.0019; main effect of time, *F*_1,30_ = 12.54, *p* = 0.0013; Fig. 2A). During the self-administration phase, nicotine animals exhibited a significant decrease in food intake compared with saline animals (*p* = 0.0094, *t* = 2.978, Student’s *t*-test). No significant difference in the total daily water intake during the nicotine ON and OFF phases was observed between nicotine and saline animals (Fig. 2B). These results confirm the previous findings that chronic nicotine intake leads to a decrease in body weight gain and feeding during nicotine use and an increase in body weight gain and feeding during nicotine cessation as the animals catch up to the control animals.

### Microstructural analysis of nicotine, food, and water self-administration events during nicotine self-administration

The microstructural analysis was performed for food, water, or nicotine events that occurred 1000 s before and after a single food, water, or nicotine event (i.e., *Y* vs. *X*). Time *t* = 0 represents the single food, water, or nicotine event. Each graph shows the average normalized probability of the total events in each 10-s bin collected during the 1000-s window surrounding the single event. Data were taken during the first day of nicotine access following a 72-h deprivation period after 2 weeks of continuous LgA to maximize the occurrence of nicotine self-administration events (George et al. 2007) and increase the statistical power of the microstructural meal pattern analysis. The nonparametric Mann–Whitney *U* test was used to compare animals that self-administered nicotine and animals that self-administered saline.

**Fig. 3:**
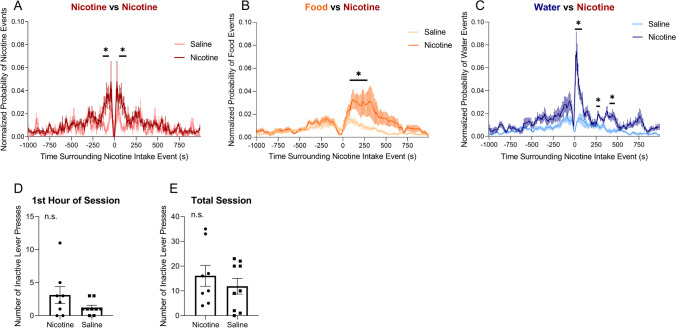
Peristimulus time histograms of drug, food, and water self-administration events that surrounded a single self-administration within a 1000-s time window. **A** Average normalized probability of nicotine (dark red) and saline (light red) intake events that surrounded a single nicotine or saline self-administration event, represented by time *t* = 0. **B** Average normalized probability of food intake events that surrounded a single nicotine (dark orange) or saline (light orange) self-administration event. **C** Average normalized probability of water intake events that surrounded a single nicotine (dark blue) or saline (light blue) self-administration event. Asterisk denotes *p* < 0.05 with Mann–Whitney test. **D** Number of inactive lever presses recorded during the first hour of the self-administration session for animals self-administering nicotine or saline. *p* = 0.1510 (Student’s *t*-test). **E** The number of inactive lever presses recorded during the total self-administration session for animals self-administering nicotine or saline. *p* = 0.4293. *N* = 8 nicotine, 9 saline

First, the effect of a single nicotine or saline self-administration event on surrounding nicotine or saline intake events was tested. Nicotine animals showed a significant increase of additional nicotine intake events surrounding a single self-administration event compared with saline animals from − 120 to − 70 s and 80 to 110 s surrounding a single nicotine event compared with saline animals (*U* = 6.5–12.0, *p* < 0.05; Fig. 3A). Next, the effect of nicotine or saline self-administration on surrounding food intake events was tested. Animals exhibited a significant increase in feeding events that followed a single nicotine self-administration event from 130 to 300 s following a single nicotine event compared with saline animals (*U* = 4.0–12.0, *p* < 0.05; Fig. 3B). The effect of nicotine or saline self-administration on surrounding water intake events was then tested. Nicotine animals exhibited a significant increase in drinking events following a single self-administration event from 0 to 90 s, 280 to 320 s, and 360 to 480 s following a single nicotine event compared with saline animals (*U* = 0–15.0, *p* < 0.05; Fig. 3C). There was no significant difference in inactive lever pressing, a measure of generalized activity, during either the first hour of the session (*p* = 0.1510, *t* = 1.513, Student’s *t*-test; Fig. 3D) or the total session (*p* = 0.4293, *t* = 0.8123, Student’s *t*-test; Fig. 3E). These results indicate that acute nicotine intake increased feeding and drinking behavior within minutes.

**Fig. 4:**
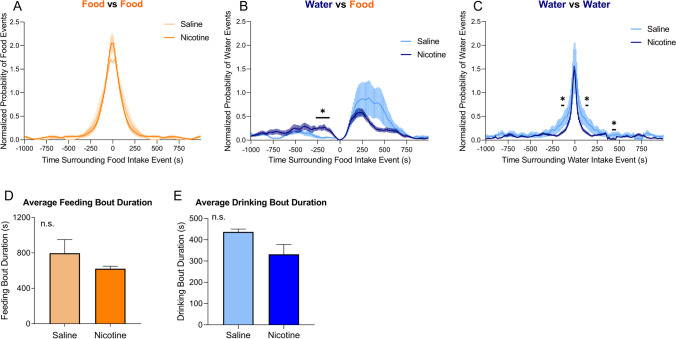
Peristimulus time histograms of food and water intake events that surrounded single food or water intake events within a 1000-s time window. **A** Average normalized probability of food intake events that surrounded a single food intake event in rats that self-administered nicotine (dark orange) or saline (light orange). **B** Average normalized probability of water intake events that surrounded a single food intake event in rats that self-administered nicotine (dark blue) or saline (light blue). **C** Average number of water intake events that surrounded a single water intake event in rats that self-administered nicotine (dark blue) or saline (light blue). Asterisk denotes *p* < 0.05 with Mann–Whitney post hoc test. **D** Average duration of feeding bout in rats that self-administered nicotine (dark orange) or saline (light orange). *p* = 0.1232 (Student’s *t*-test). **E** Average duration of drinking bout in rats that self-administered nicotine (dark blue) or saline (light blue). *p* = 0.2193 (Student’s *t*-test). Nicotine (*n* = 8), saline (*n* = 3)

PSTH were generated to compare the effects of nicotine vs. saline self-administration on feeding and drinking bouts. There was no significant difference in feeding bout peaks between nicotine and saline self-administration animals (*U* = 1.0–10.0, *p* > 0.05; Fig. 4A). However, nicotine animals exhibited a significant decrease in water intake following a single feeding event, with a significant increase in water intake from − 260 to − 130 s prior to a single food intake event in nicotine animals compared with saline animals (*U* = 0–1.0, *p* < 0.05; Fig. 4B). Nicotine animals also showed a significant decrease in drinking events surrounding a single water intake event from − 110 to − 100 s prior to a single water intake event and 100–110 and 400–460 s after the intake event (*U* = 2.0–3.0, *p* < 0.05; Fig. 4C). Next, changes in the duration of each food and drinking bout were analyzed. There was no significant difference in feeding bout duration between nicotine and saline animals (*p* = 0.1232, *t* = 1.723, Student’s *t*-test; Fig. 4D). No significant difference in the duration of a water bout was found between nicotine and saline animals (*p* = 0.2193, *t* = 1.320, Student’s *t*-test; Fig. 4E). These results show that while feeding bout duration does not differ between nicotine-administering and saline-administering animals; nicotine-administering animals display decreased drinking bout duration and decreased water intake in relation to feeding bouts.

### Microstructural analysis of nicotine, food, and water intake events during passive nicotine or saline administration

To test the pharmacological effects of nicotine on feeding and drinking independently of the operant behavior producing nicotine self-administration, saline or nicotine was passively administered intravenously using the MedPC program. The animals were given one infusion of 0.9% saline, one infusion of nicotine (0.03 mg/kg/infusion), or three infusions of nicotine (0.03 mg/kg/infusion) and then allowed to nosepoke for food and water but had no access to the nicotine levers. These doses were chosen to represent no nicotine intake, a single nicotine intake event, and a “burst” of nicotine intake, respectively. As shown in Fig. 3A, animals self-administer an average burst of 3 nicotine infusions, so this number was used in the passive infusion experiments to replicate this phenomenon. These infusions were administered once every hour for 8 h in order to generate enough data points for statistical analysis. Nonparametric Kruskal–Wallis test with Dunn’s multiple comparisons post hoc was used to compare effects between each group.

**Fig. 5:**
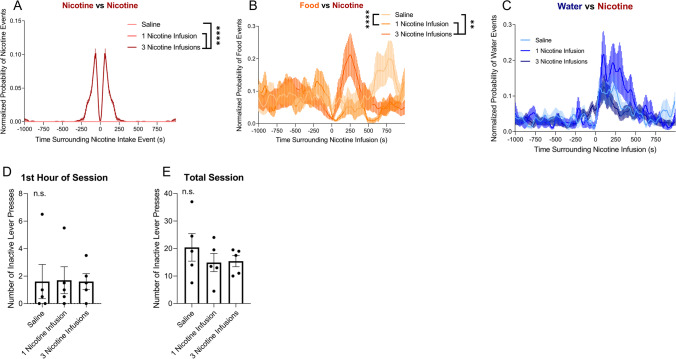
Peristimulus time histograms of nicotine, food, and water intake events that surrounded passive administration events within a 1000-s time window. **A** Average normalized probability of 1 saline infusion (pink), 1 nicotine infusion (red), and 3 nicotine infusions (maroon). **B** Average normalized probability of food intake events in rats following three nicotine infusions (dark orange), one nicotine infusion (orange), or one saline infusion (light orange). **C** Average normalized probability of water intake events in rats following three nicotine infusions (dark blue), one nicotine infusion (blue), or 1 saline infusion (light blue). **p < 0.01, ****p < 0.0001, Kruskal–Wallis and Dunn’s multiple comparisons post hoc. *N* = 7 per group. **D** Number of inactive lever presses recorded during the first hour of the self-administration session for animals receiving 1 saline infusion, 1 nicotine infusion, or 3 nicotine infusions. *p* = 0.9709 (one-way repeated measures ANOVA). **E** Number of inactive lever presses recorded during the total self-administration session for animals receiving 1 saline infusion, 1 nicotine infusion, or 3 nicotine infusions. *p* = 0.3703 (one-way repeated measures ANOVA). *N* = 5 per group

Figure 5A shows the average normalized probability of nicotine intake events surrounding for the passive infusions administered to each group — one saline infusion, one nicotine infusion, or 3 nicotine infusions. Animals given three nicotine infusions displayed a significant increase in nicotine events surrounding a single infusion compared with the other groups (*p* < 0.0001) between animals receiving 3 nicotine infusions compared with both one nicotine infusion and one saline infusion. The effect of passive administration events on surrounding food intake events was tested next. The Kruskal–Wallis test revealed a significant difference between treatment groups on food intake events surrounding the passive infusions (*p* < 0.0001; Fig. 5B). Next, the effect of passive administration events on surrounding water intake events was tested. There was no significant difference between groups (*p* = 0.1199; Fig. 5C). There was no significant difference in inactive lever pressing, a measure of generalized activity, during either the first hour of the session (*p* = 0.9709, *F* = 0.0146; Fig. 5D) or the total session (*p* = 0.3703, *F* = 1.104; Fig. 5E). These results indicate that passive nicotine administration similarly increased food and water self-administration compared with nicotine self-administration.

**Fig. 6:**
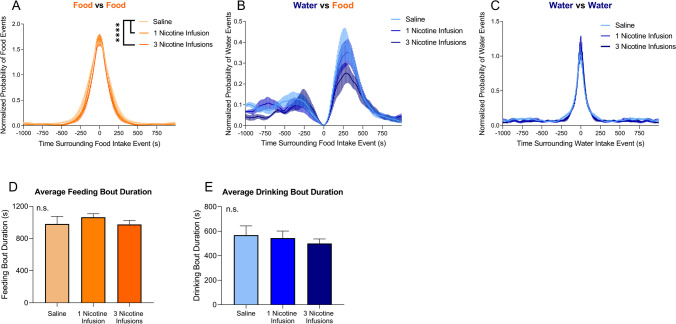
Peristimulus time histograms of food and water intake events that surrounded single food or water intake events within a 1000-s time window. **A** Average normalized probability of food intake events compared with a single food intake event in rats that were given three nicotine infusions (dark orange), one nicotine infusion (orange), or one saline infusion (light orange). **B** Average normalized probability of water intake events compared with a single food intake event in rats that were given three nicotine infusions (dark blue), one nicotine infusion (blue), or one saline infusion (light blue). **C** Average normalized probability of water intake events compared with a single water intake event in rats that were given three nicotine infusions (dark blue), one nicotine infusion (blue), or one saline infusion (light blue). ****p < 0.0001, Kruskal–Wallis with Dunn’s multiple comparisons post hoc. **D** Average duration of feeding bout in rats that self-administered nicotine (dark orange) or saline (light orange). *p* = 0.5838 (one-way ANOVA). **E** Average duration of drinking bout in rats that self-administered nicotine (dark blue) or saline (light blue). *p* = 0.7154 (one-way ANOVA). *N* = 5 per group

PSTH were generated to compare the effects of each passive administration treatment on the average normalized probability of feeding and drinking bouts. There was a significant difference in feeding bouts between the saline group and both nicotine infusion groups (*p* < 0.0001; Fig. 6A). There was no difference in water intake surrounding a single food intake event or drinking bouts between treatment groups (Fig. 6B and C). Changes in feeding and drinking bout duration were also analyzed by taking the derivatives of the PSTH curves and measuring the time between the two points where the first derivative was 0. No significant difference in feeding bout duration was observed between treatment groups (Fig. 6D). No significant difference in drinking bout duration was observed between treatment groups (Fig. 6E). These results show that passive administration alters feeding bout curve shape but not duration, but there is no difference between treatment groups in drinking bouts or water intake surrounding a single food intake event.

## Discussion

This study tested the hypothesis that on both a macrostructural and a microstructural level, nicotine will decrease food and water intake. Consistent with previous findings, long-term self-administration of nicotine elicited a decrease in animals’ body weight gain and daily total food intake and increase in body weight gain and food intake during abstinence. However, and unexpectedly, nicotine self-administration did not produce any sign of a decrease in food intake within minutes of nicotine administration. Instead, nicotine increased feeding and drinking events within minutes compared with saline self-administration. This increase was also observed after passive administration of nicotine.

The macrostructural analysis data showed that following chronic nicotine self-administration, animals exhibited a 5% decrease in body weight gain and 10% decrease in food intake compared with saline animals. They also exhibited a 20% increase in food intake during nicotine abstinence compared with when nicotine was available. During each cycle of the intermittent access paradigm, the body weight stopped increasing or even decreased in nicotine animals until the end of the self-administration (ON) phase and robustly increased by the end of the abstinence (OFF) phase. These results support our hypothesis that nicotine intake decreases feeding and weight gain and are consistent with previous studies that reported a decrease in body weight gain during chronic nicotine self-administration and a robust increase following nicotine cessation (Aubin et al. 2012; L. L. Bellinger et al. 2010; Grunberg et al. 1986; Rupprecht et al. 2018; Winders & Grunberg 1990). The increase in food intake in nicotine animals during abstinence from nicotine is consistent with previous studies in both animals and humans (Bowen et al. 1986; Faraday et al. 2001; Filozof et al. 2004; Stamford et al. 1986). We found that water intake did not change with chronic nicotine use, which is consistent with previous studies that reported that chronic nicotine did not alter water intake in animals (Clarke & Kumar 1984; Levin et al. 1987).

Microstructural analysis of behavior using PSTH is a relatively novel approach within the field of nicotine and drug self-administration. PSTH are commonly used to represent electrophysiological data, where neuronal firing rate (in our case, behavioral events) is measured surrounding a stimulus (single event of interest, in our case a single nicotine, food, or water intake event) (Gerstein & Kiang 1960; Moore et al. 1966). One advantage of such analysis is that it provides greater temporal resolution (seconds) and may reveal short-lasting effects that can be masked using traditional behavioral analysis. This is particularly relevant to studying feeding behavior, which is commonly measured on an hour-day timescale. As PSTH graph behaviors surrounding an event of interest, they also allow us to evaluate the temporal relationship between behavioral events, further refining the quantitative analysis of each event and providing additional information regarding the potential directionality of causal effects.

Microstructural analyses of behavior during nicotine self-administration showed that rats exhibited a four-fold increase in the normalized probability of food intake and a ten-fold increase in the normalized probability of water intake within 10 min of nicotine self-administration compared with saline-self-administration. PSTH data used the normalized probability of intake behavior, rather than average intake events, to control for variability between individual animals’ intakes. These results are particularly striking considering that a similar microstructural analysis conducted by Cooper and van der Hoek of feeding behavior after administration of cocaine, another psychostimulant and anorectic substance, was associated with a complete suppression of feeding, with the duration of suppression proportional to the dose (Cooper & van der Hoek 1993). Additionally, a recent study by Romero et al. employed microstructural analysis techniques to analyze the reinforcer-enhancing effects of nicotine on food-seeking behavior (Romero et al. 2018). They found that lower doses of nicotine administered via injection increased the rate of food-seeking bouts. While their study looked at reinforced food-seeking behavior and not ad lib feeding during self-administration, it is possible that our results may also be due to nicotine use enhancing the rewarding value of food. Our results do suggest, though, that the increased feeding observed minutes after acute nicotine intake is not a common property of psychostimulant and anorectic agents, but that it is specific to nicotine.

An alternate explanation is that this phenomenon may be nonspecific and attributable to a general increase in operant behavior. When we compared responses at the inactive lever, a commonly used measure for nonspecific activity within the operant chamber (Lu et al. 2004; Ranaldi et al. 2011; Richards et al. 2008; Shaham et al. 1997), we did not observe any differences between the animals. If the increase in behavior was due to a concomitant increase in activity following nicotine intake, we would have expected to see a significant increase in inactive lever presses in the nicotine-administering animals. However, the increase in food intake was not observed following saline self-administration, so it may be possible that nicotine-enhanced activity could contribute to, but is not solely responsible for, the increase in acute feeding and drinking behavior.

To date, there are no studies which have examined effects of acute nicotine intake on short-term changes in feeding or drinking behavior. However, these results may be supported through studies which have examined nicotine’s effects on metabolic or neuronal changes. Bouros et al. found that plasma ghrelin, commonly known as a “hunger hormone” was increased at 2, 5, and 15 min following cigarette smoking in human smokers and nonsmokers (2006). Additionally, nicotine has been shown to activate agouti-related protein (AgRP) neurons within the arcuate nucleus of the hypothalamus, a neuronal population which is known to stimulate feeding behavior (Huang et al. 2011), providing a possible mechanism by which nicotine may be exerting acute anti-anorectic effects.

The increase in the probability of feeding and drinking behavior following nicotine self-administration was also seen in animals following passive administration of nicotine. Rats given three nicotine infusions exhibited a three-fold increase in food intake within 5–6 min compared with rats given one saline or one nicotine infusion. All treatment groups displayed an increase in water intake compared to baseline. These results confirm that the increase in food and water intake is nicotine-specific, rather than a nonspecific effect of operant behavior. Furthermore, analysis of the normalized probability of intake behavior showed that although there was a small sample size, there was low variability in the data, and the effects seen were consistent within the treatment groups. The increase in food intake was also observed following three, but not one passive infusions of nicotine demonstrating that this effect is dose-dependent. Considering that rats also self-administered on average 3 consecutive infusions of nicotine during a nicotine bout, it suggests that the pharmacological effect of nicotine at an approximate dose of 0.09 mg/kg was responsible for the increase in food intake. A study by O’Dell et al. showed differences in average meal size and average meal duration between rats self-administering 0.03 mg/kg/infusion nicotine and 0.06 mg/kg/infusion nicotine (2007), suggesting that 0.06–0.09 mg/kg is likely to be a critical dose range for nicotine-induced increase in feeding.

Calculating feeding bout duration by analyzing the derivatives of the PSTH curves did not reveal a significant difference between nicotine or saline animals; however, passive nicotine infusion did show altered structure of the feeding bout curve. This suggests that nicotine use may change the overall structure of food intake bouts, and that the nicotine-induced fragmentation of feeding bouts may contribute to the long-term changes in feeding and body weight. In fact, it has been shown that long-term nicotine use does alter meal patterns (L. Bellinger et al. 2003; L. L. Bellinger et al. 2005, 2010). Our data may provide additional insight into the structure of this meal pattern dysregulation. Drinking bout duration was not changed following either self-administration or passive administration of either nicotine or saline. Nicotine animals also exhibited a 20% decrease in water intake following a food intake event during self-administration. However, this decrease in water intake following a food intake event was not observed in animals following passive nicotine administration. One potential explanation for this discrepancy is that this effect was nonspecific and caused by changes in operant behavior. Studies measuring nicotine’s effects on water intake have conflicting reports; while some studies report no long-term change in water intake as we described above, others report a transient decrease in water intake (although still at the hour-day scale) (Clarke & Kumar 1984; Levin et al. 1987; Rupprecht et al. 2018).

The results of the current report challenge the dogma that the initial pharmacological effect of nicotine is solely anorectic and instead suggest that the initial pharmacological properties of nicotine paradoxically may be anti-anorectic. While the increase in food intake in terms of calories is unlikely to produce a large effect on homeostatic regulation of feeding, it is highly significant from a pharmacological perspective and for drug development studies, considering that this effect occurs during the time that blood and brain nicotine concentrations are at peak levels. For instance, it is possible that the initial pharmacological effect of activation of nicotine receptors leads to increases in food intake while the lasting desensitization effect of nicotine receptors may lead to the prolonged anorexic effect of nicotine (Picciotto et al. 2008). The present results are also consistent with a study by Perkins et al. that showed that smokers actually reported an increase in caloric intake after acute nicotine exposure (1991). The opposite effects of acute and chronic nicotine on feeding behavior may be explained by Perkins’ theory that nicotine alters the homeostatic set point of body weight. Perkins and colleagues showed that although body weight decreases with chronic nicotine use in humans, eating increases following acute nicotine intake (Perkins et al. 1991). The metabolic effects of nicotine may contribute to a change in body weight set point, with consequent compensatory changes in caloric intake (Perkins 1992). The present results are consistent with Perkins’s theory and provide evidence that nicotine interacts with body metabolism to elicit an acute increase in food intake while also leading to a decrease in body weight over time.

In summary, the present study found that nicotine first produces increases in food intake before producing long-term anorexigenic effects. These results challenge the dogma that nicotine is solely an anorexigenic drug and suggest that further studies are needed to understand the cellular and molecular mechanisms of both the immediate and long-term effects of nicotine on feeding, motivation, and receptor activation/desensitization. A better understanding of the acute and long-term effects of nicotine on feeding behavior may contribute to the development of alternative strategies to treat tobacco use disorder and obesity.
